# Prevalence of Erectile Dysfunction in Malagasy Patients With Diabetes Mellitus and Its Associations With Atherosclerotic Cardiovascular Diseases: A Cross‐Sectional Study

**DOI:** 10.1002/edm2.70098

**Published:** 2025-08-25

**Authors:** Sitraka Angelo Raharinavalona, Tafitarilova Dorland Ranjandriarison, Thierry Razanamparany, Romuald Randriamahavonjy, Andrianirina Dave Patrick Rakotomalala

**Affiliations:** ^1^ Cardiovascular Diseases and Internal Medicine Department Soavinandriana Hospital Center Antananarivo Madagascar; ^2^ Obstetrics and Gynecology Department Soavinandriana Hospital Center Antananarivo Madagascar; ^3^ Endocrinology Department Joseph Raseta Befelatanana University Hospital Center Antananarivo Madagascar

**Keywords:** atherosclerotic cardiovascular disease, diabetes mellitus, erectile dysfunction, Madagascar

## Abstract

**Objectives:**

Our study aims to determine the prevalence of erectile dysfunction (ED) and its associations with atherosclerotic cardiovascular disease (ASCVD) in Malagasy patients with diabetes mellitus (DM).

**Methods:**

This cross‐sectional study was conducted over a period of 3 years at the Soavinandriana Hospital Center. Erectile function was assessed using the International Index of Erectile Function 5‐item version (IIEF‐5) questionnaire, with a score of less than 22 indicating ED. The presence of ASCVD was confirmed in cases where carotid atherosclerosis (CAS), lower limb arteriopathy (LLA), coronary heart disease (CHD) and/or ischaemic stroke were present.

**Results:**

The study population included 305 patients diagnosed with diabetes mellitus (DM). The prevalence of erectile dysfunction (ED) was 73.4%. According to the bivariate analysis, the risk factors for ED were age ≥ 55 years (odds ratio [OR] 12.0 (6.34–23.4)), hypertension (OR 6.02 (3.27–11.3)), physical inactivity (OR 8.86 (4.85–16.6)), smoking (OR 2.53 (1.32–5.09)), dyslipidemia (OR 4.11 (2.33–7.32)), type 2 DM (OR 8.80 (1.53–91.0)) and diabetes duration ≥ 10 years (OR 2.24 (1.11–4.87)), renin‐angiotensin‐aldosterone system blockers (OR 6.27 (3.43–11.6)), calcium‐channel blockers (OR 3.01 (1.69–5.48)), diuretics (OR 2.14 (1.06–4.66)) and beta‐blockers (OR 4.55 (1.85–13.5)). After adjusting for age, hypertension, physical inactivity, smoking and dyslipidemia, ED was significantly associated with ASCVD (OR 1.88 (1.01–3.69)), and CAS (OR 1.89 (1.03–3.22)). Adjusting for age, type and duration of DM, ED was found to be significantly associated with ASCVD (OR 1.91 (1.01–3.58)) and CAS (OR 2.31 (1.11–4.85)). However, there was no statistically significant association between ED, LLA, CHD and ischaemic stroke.

**Conclusion:**

ED was very common and may be a predictor of ASCVD in patients with DM, particularly CAS. Consequently, the presence of ED should prompt the search for ASCVD, and vice versa.

## Graphical abstract

The erectile dysfunction (ED) was very common and may be a predictor of atherosclerotic cardiovascular disease (ASCVD) in Malagasy patients with diabetes mellitus. Consequently, the presence of ED should prompt the search for ASCVD, and vice versa.

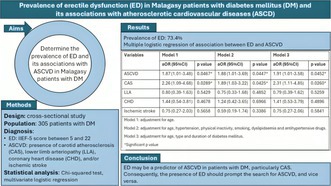

## 1 Background

Erectile dysfunction (ED) is defined as the persistent or recurrent inability of a man to obtain or maintain an erection sufficient for satisfactory sexual intercourse [1]. It significantly impacts the quality of life of patients, their partners and even their families [2, 3].

In patients with diabetes mellitus (DM), ED is particularly common. The global prevalence is 52.5% (95% confidence interval (CI) 48.8%–56.2%) [4]. In Africa, it has risen to 71.45% (CI 60.22%–82.69%) [5]. In a previous study in Antananarivo, Madagascar, 72.7% of diabetic patients had ED [6]. The prevalence of ED in patients with DM is approximately 3.5 times greater than that in patients without DM [7]. The pathophysiology of ED in DM patients is multifactorial, including vascular, hormonal and neurological damage [8]. Risk factors for ED in patients with DM include age, duration of DM, physical inactivity, peripheral neuropathy, peripheral vascular diseases and low testosterone levels [9]. Additionally, ED is significantly associated with other cardiovascular risk factors and a high risk of cardiovascular events [10, 11]. Health literacy plays an important role in preventing cardiovascular disease, as well as ED in patients with DM [12].

In Madagascar, ED is often underdiagnosed in both patients with and without DM, considered a taboo subject and lacks available data [6, 13]. To address this knowledge gap, the present study was conducted with the objective of determining the prevalence of ED and specifically its associations with atherosclerotic cardiovascular disease (ASCVD) in patients with DM seen at the Soavinandriana Hospital Center to enhance their care.

## 2 Materials and Methods

### 2.1 Study Design and Setting

A cross‐sectional study was conducted over a period of 3 years from July 1, 2020, to September 30, 2023, at the Department of Cardiovascular Diseases and Internal Medicine of the Soavinandriana Hospital Center (Military Hospital), Antananarivo, the capital of Madagascar. These departments are recognised as key references for the management of cardiovascular, cerebrovascular, endocrine, metabolic and internal medicine diseases in Madagascar.

### 2.2 Study Population

The study population consisted of patients with diabetes mellitus who were receiving external treatment during the period of study. The study included all patients aged 18 years or older, with either known or newly diagnosed diabetes, who completed and returned the International Index of Erectile Function 5‐item version (IIEF‐5) questionnaire, and who benefited from research on the main ASCVDs such as carotid atherosclerosis (CAS), lower limb arteriopathy (LLA), coronary heart disease (CHD) and ischaemic stroke. Patients with an uninterpretable IIEF‐5 score (1 to 4 points), those with neurological or mental disorders that made responding difficult, those who refused to participate and those with incomplete data were excluded from the study.

The population sampling was thorough and exhaustive. The sample size was determined using a single population proportion formula: *n* = (Z1‐α/2)^2^ P (1‐P)/d^2^; based on the assumptions of a 95% confidence level (Z1‐α/2 = 1.96), 5% margin of error (d) and 72.7% prevalence (P) of ED according to the study by Raharinavalona et al. [6]. The sample size for the study was determined to be 305 patients with DM who consented to participate.

### 2.3 Clinical and Laboratory Data

The data were accessed by two investigators from July 1, 2020, to September 30, 2023. The parameters studied included sociodemographic characteristics (age, education level and residence), associated cardiovascular risk factors (hypertension, physical inactivity, overweight or obesity, smoking and dyslipidemia), characteristics of DM (type 1 and 2, duration, medication, glycated haemoglobin or Hb A1c), ASCVD (CAS, LLA, CHD and ischaemic stroke) and ED (severe, moderate, mild to moderate, mild and no ED).

Hypertension was confirmed by a blood pressure ≥ 140/90 mmHg, based on an average of ≥ 2 measurements obtained on ≥ 2 occasions or the use of antihypertensive medication. Body mass index (BMI) was calculated as weight in kilograms (kg) divided by height square in meters (m^2^). Overweight and obesity are defined by a BMI ≥ 25 kg/m^2^ and ≥ 30 kg/m^2^, respectively. Patients with low‐density lipoprotein cholesterol (LDLc) levels outside the targets recommended by the European Society of Cardiology [14] or those who were taking a lipid‐lowering agent were considered to have dyslipidemia.

The diagnosis of DM was based on the criteria outlined by the American Diabetes Association, including fasting plasma glucose ≥ 126 mg/dL (7.0 mmol/L), 2‐h plasma glucose ≥ 200 mg/dL (11.1 mmol/L) during the 75‐g oral glucose tolerance test, Hb A1c ≥ 6.5% (48 mmol/mol) or classic symptoms of hyperglycaemia and random plasma glucose ≥ 200 mg/dL (11.1 mmol/L) [15]. Type 1 DM was considered in a young patient (< 35 years) of normal weight or skinny, confirmed by the positivity of at least one autoantibody. On the other hand, type 2 DM was associated with patients over 35 years of age, overweight/obesity, a family history of type 2 DM and the absence of obvious secondary causes such as diseases of the exocrine pancreas or drug‐ or chemical‐induced diabetes. Uncontrolled DM was defined as Hb A1c ≥ 7%, as measured using high‐performance liquid chromatography.

The presence of ASCVD was confirmed in cases of CAS, LLA, CHD and/or ischaemic stroke. CAS was defined as the presence of carotid plaque or diffuse thickening of the carotid wall (≥ 1.1 mm) [16]. LLA, in either its stenosing atherosclerosis form or in the absence thereof, or medical calcinosis, was confirmed by arterial Doppler ultrasound. The diagnosis of CHD is made on the basis of the presence or absence of typical or atypical chest pain and/or other atypical signs, the presence of a Q wave of necrosis and negative T in one or more territories defined on the electrocardiogram, and segmental akinesia or hypokinesia on echocardiography [17, 18]. The diagnosis of ischaemic stroke is made in the presence of focal neurological signs of sudden onset, including motor and/or sensory deficits, and is confirmed by the presence of parenchymal hypodensity corresponding to one or more affected arterial territories(s) on brain scan [19].

Erectile function was assessed using the IIEF‐5 questionnaire. Patients with a score between22 and 25 were classified as having normal erectile function, while those with a score below 22 were classified as severe (5–7), moderate (8–11), mild to moderate (12–16) or mild (17–21) ED [20].

### 2.4 Statistical Analysis

The data were collected from patient medical records using a predetermined survey form. Subsequently, the data underwent analysis using Epi Info version 3.5.4 software (United States Centers for Disease Control and Prevention in Atlanta, Georgia). The qualitative and quantitative variables were expressed as percentages and medians (interquartile ranges (IQR) 25%–75%), respectively. Chi‐squared test with a significance threshold of less than 0.05 and odds ratio (OR) were used to identify factors associated with ED. Logistic regression analysis was used to determine the associations between ED and ASCVD.

## 3 Results

During the study period, 305 patients who met the eligibility criteria were included. The prevalence of ED was 73.4%, with 19.7% of cases classified as severe (see Table 1). The median age of the patients was 64 years (IQR 55.5–69). The predominant cardiovascular risk factors were hypertension and dyslipidemia in 76.7% and 67.2% of the patients, respectively. Type 2 DM was prevalent in 97.4% of patients, with a median duration of 5 years (IQR 2–10.5). Almost three quarters of patients (74.1%) had uncontrolled DM. According to the bivariate analysis, the risk factors for ED were age ≥ 55 years (OR 12.0 (6.34–23.4)), hypertension (OR 6.02 (3.27–11.3)), physical inactivity (OR 8.86 (4.86–16.6)), smoking (OR 2.53 (1.32–5.09)), dyslipidemia (4.11 (2.33–7.32)), type 2 DM (OR 8.8 (1.53–91)) diabetes duration ≥ 10 years (OR 2.24 (1.11–4.87)), the use of renin‐angiotensin‐aldosterone system (RAAS) blockers (OR 6.27 (3.43–11.6)), calcium‐channel blockers (OR 3.01 (1.69–5.48)), diuretics (OR 2.14 (1.06–4.66)) and beta‐blockers (OR 4.55 (1.85–13.5)). In 39.4% of our patients, those with diabetes suffered from at least one ASCVD. According to the bivariate analysis, ASCVD was significantly associated with ED. Table 2 summarises the general characteristics of the study population.

**TABLE 1:** Distribution of patients according to their erectile function.

Variables	Numbers	Percentage
Normal erectile function	81	26.6
Erectile dysfunction	224	73.4
Severe	60	19.7
Moderate	61	20.0
Mild to moderate	53	17.3
Mild	50	16.4

**TABLE 2:** General characteristics of the study population.

Variables	Total (*N* = 305)	Without ED (*n* = 81)	With ED (*n* = 224)	OR (95% CI)	*p*
Age (years)					
Median, (IQR)	64 (55.5–69)	52 (44–61)	66 (59–70)	—	< 0.0001*
≥ 55, *n* (%)	231 (75.7)	32 (39.5)	199 (88.8)	12.0 (6.34–23.4)	< 0.0001*
Education level					
Primary, *n* (%)	57 (18.7)	11 (13.6)	46 (20.5)	1.64 (0.78–3.72)	0.1114
Secondary, *n* (%)	123 (40.3)	33 (40.7)	90 (40.2)	Reference	
University, *n* (%)	125 (41.0)	37 (45.7)	88 (39.3)	0.77 (0.44–1.33)	0.1917
Residence					
Urban, *n* (%)	205 (67.2)	52 (64.2)	153 (68.3)	1.43 (0.39–1.98)	0.1437
Rural, *n* (%)	100 (32.8)	29 (35.8)	71 (31.7)	Reference	
Cardiovascular risk factors					
Hypertension, *n* (%)	234 (76.7)	41 (50.6)	193 (86.2)	6.02 (3.27–11.3)	< 0.0001*
Physical inactivity, *n* (%)	201 (65.9)	24 (29.6)	177 (79.0)	8.86 (4.85–16.6)	< 0.0001*
Overweight or obesity, *n* (%)	149 (48.9)	49 (60.5)	100 (44.6)	0.55 (0.31–1.24)	0,0538
Smoking, *n* (%)	97 (31.8)	15 (18.5)	82 (36.6)	2.53 (1.32–5.09)	0.0011*
Dyslipidemia, *n* (%)	205 (67.2)	35 (43.2)	170 (75.9)	4.11 (2.33–7.32)	< 0.0001*
DM characteristics					
Type 1, *n* (%)	8 (2.6)	6 (7.4)	2 (0.9)	Reference	
Type 2, *n* (%)	297 (97.4)	75 (92.6)	222 (99.1)	8.80 (1.53–91.0)	0.0029*
Median duration, years (IQR)	5 (2–10.5)	3 (0–8)	6 (3–11)	—	0.0002*
Duration ≥ 10 years, *n* (%)	75 (24.6)	12 (14.8)	63 (28.1)	2.24 (1.11–4.87)	0.0108*
Median Hb A1c, % (IQR)	8.3 (6.9–10.5)	8.7 (7.2–10.4)	8.2 (6.8–10.8)	—	0.7676
Hb A1c ≥ 7%, *n* (%)	226 (74.1)	65 (80.2)	161 (71.9)	0.63 (0.32–1.21)	0.0907
ASCVD, *n* (%)	137 (44.9)	23 (28.4)	114 (50.9)	2.61 (1.46–4.74)	< 0.0001*
CAS, *n* (%)	88 (28.9)	13 (16.0)	75 (33.5)	2.62 (1.33–5.52)	< 0.0011*
LLA, *n* (%)	70 (23.0)	16 (19.8)	54 (24.1)	1.29 (0.66–2.59)	0.2623
CHD, *n* (%)	43 (14.1)	6 (7.4)	37 (16.5)	2.46 (1.00–7.45)	0.0287*
Ischaemic stroke, *n* (%)	32 (10.5)	6 (7.4)	26 (11.6)	1.64 (0.62–5.06)	0.2012
Antihypertensive drugs					
RAAS blockers, *n* (%)	228 (74.8)	38 (46.9)	190 (84.8)	6.27 (3.43–11.6)	< 0.0001*
CC blockers, *n* (%)	145 (47.5)	23 (28.4)	122 (54.5)	3.01 (1.69–5.48)	< 0.0001*
Diuretics, *n* (%)	73 (23.9)	12 (14.8)	61 (27.2)	2.14 (1.06–4.66)	0.0158*
Beta‐blockers, *n* (%)	66 (21.6)	6 (7.4)	60 (26.8)	4.55 (1.85–13.5)	< 0.0001*
Antidiabetics drugs					
OAD, *n* (%)	180 (59.0)	51 (63.0)	129 (57.6)	0.79 (0.45–1.38)	0.2392
Insulin, *n* (%)	51 (16.7)	14 (17.3)	37 (16.5)	0.94 (0.46–2.02)	0.4988
OAD and insulin, *n* (%)	62 (20.3)	13 (16.0)	49 (21.8)	1.15 (0.76–2.38)	0.4781

Abbreviations: ASCVD, atherosclerotic cardiovascular disease; CAS, carotid atherosclerosis; CC, calcium‐channel; CHD, coronary heart disease; CI, confidence interval; DM, diabetes mellitus; ED, erectile dysfunction; Hb A1c, glycated haemoglobin; IQR, interquartile range; LLA, lower limbs arteriopathy; OAD, oral antidiabetics; OR, odds ratio; RAAS, renin‐angiotensin‐aldosterone system.

* Significant *p* value.

After adjustments for age, hypertension status, physical inactivity status, smoking status, dyslipidemia status and antihypertensive drugs, multivariate analysis revealed a significant association between ED status and ASCVD (OR 1.88 (1.01–3.69)), and CAS (OR 1.89 (1.03–3.22)). Table 3 presents the findings of multiple logistic regression analysis of the association between ED and macrovascular complications.

**TABLE 3:** | Multiple logistic regression of association between erectile dysfunction and atherosclerotic cardiovascular disease.

Variables	Model 1		Model 2		Model 3	
	aOR (95% CI)	*p*	aOR (95% CI)	*p*	aOR (95% CI)	*p*
ASCVD	1.87 (1.01–3.48)	0.0467*	1.88 (1.01–3.69)	0.0447*	1.91 (1.01–3.58)	0.0452*
CAS	2.26 (1.09–4.68)	0.0289*	1.89 (1.03–3.22)	0.0425*	2.31 (1.11–4.85)	0.0260*
LLA	0.80 (0.39–1.63)	0.5429	0.75 (0.33–1.68)	0.4852	0.79 (0.39–1.62)	0.5259
CHD	1.44 (0.54–3.81)	0.4678	1.24 (0.42–3.65)	0.6966	1.41 (0.53–3.79)	0.4896
Ischaemic stroke	0.75 (0.27–2.03)	0.5658	0.59 (0.19–1.74)	0.3386	0.75 (0.27–2.06)	0.5841

*Note:* Model 1: adjustment for age. Model 2: adjustment for age, hypertension, physical inactivity, smoking, dyslipidaemia and antihypertensive drugs. Model 3: adjustment for age, type and duration of diabetes mellitus.

Abbreviations: aOR, adjusted odds ratio; ASCVD, atherosclerotic cardiovascular disease; CAS, carotid atherosclerosis; CHD, coronary heart disease; CI, confidence interval; LLA, lower limbs arteriopathy.

* Significant *p* value.

The severity of ED was found to be significantly worse in cases involving all forms of ASCVD. Associations between ED classification and ASCVD are summarised in Table 4.

**TABLE 4:** Association between erectile dysfunction classification and atherosclerotic cardiovascular disease.

Variables	Without ED (*n* = 81)	Mild ED (*n* = 50)	Mild‐to‐moderate ED (*n* = 53)	Moderate ED (*n* = 61)	Severe ED (*n* = 60)	*p*
ASCVD, *n* (%)	23 (29.1)	12 (23.1)	25 (47.2)	28 (45.9)	49 (81.7)	< 0.0001*
CAS, *n* (%)	13 (16.5)	3 (5.8)	18 (34.0)	19 (31.1)	35 (58.3)	< 0.0001*
LLA, *n* (%)	16 (20.3)	11 (21.2)	10 (18.9)	11 (18.0)	22 (36.7)	0.0867
CHD, *n* (%)	6 (7.6)	1 (1.9)	9 (17.0)	8 (13.1)	19 (31.7)	0.0001*
Ischaemic stroke, *n* (%)	6 (7.6)	0 (0)	4 (7.5)	3 (4.9)	19 (31.7)	< 0.0001*

Abbreviations: aOR, adjusted odds ratio; ASCVD, atherosclerotic cardiovascular disease; CAS, carotid atherosclerosis; CHD, coronary heart disease; CI, confidence interval; ED, erectile dysfunction; LLA, lower limbs arteriopathy.

* Significant *p* value.

## 4 Discussion

In the present study, the prevalence of ED was 73.4%. This finding was consistent with the results of studies conducted in Antananarivo [6], Ethiopia [21] and Ivory Coast [22], which reported prevalence rates of 72.7%, 72.2%, 72.9% and 74.1%, respectively. Conversely, ED was more common in studies conducted in Egypt (80%) [23], Sudan (80%) [24], Gabon (82.6%) [25], Ethiopia (84.3%) [26] and Morocco (88%) [27]. Its prevalence was lower in studies conducted in Germany (23%) [28], the United States of America (30%) [29], India (59.38%) [30], Sri Lanka (62.9%) [31] and China (66.7%) [32]. This discrepancy in prevalence can be attributed to variations in the studied population, cultural differences across countries and the questionnaires with their translations which facilitate the diagnosis of ED. Nevertheless, ED is more common, especially in African countries, including ours.

In the present study, as in several others [21, 28, 33], advanced age was identified as an independent risk factor for ED. Indeed, elderly diabetic subjects most often present with other risk factors for ED, including cardiovascular risk factors, long duration of diabetes and chronic degenerative complications [34, 35, 36]. With advancing age, male subjects demonstrate a reduced response to fantasy and visual stimuli. The attainment of an erection necessitates direct stimulation of the penis, often requiring prolonged stimulation [37]. The educational levels and residences of our patients were not found to be significantly associated with ED. This observation aligns with findings of studies conducted in Ethiopia [21, 38], Sudan [24] and China [32].

The patient population in this study exhibited a number of additional cardiovascular risk factors, as previously documented in the extant literature [6, 27, 33]. The present study demonstrated that hypertension, dyslipidaemia, physical inactivity and smoking were risk factors for ED. In accordance with these findings, numerous researchers have identified hypertension as a predictive factor of ED [25, 31, 33]. The findings of studies by Omar et al. [24] and Milama et al. [25] further substantiated the deleterious role of lipid profile abnormalities in the manifestation of ED. Two African studies have demonstrated a significant association between physical inactivity and ED [26, 39]. In the study by Chaudhary et al., smoking was identified as a risk factor for ED [32]. Consequently, the management of other cardiovascular risk factors remains essential for improving erectile function in diabetic patients. Moreover, there is insufficient evidence to ascertain the impact of antihypertensive drugs on sexual function [40, 41].

The present study has found that type 2 DM was a risk factor for ED. This finding aligns with the observations reported by Nisahan et al. [31]. Indeed, type 2 diabetic patients are often elderly and have other cardiovascular risk factors and chronic degenerative complications that could further promote the occurrence of ED. In accordance with the extant literature, our study corroborates the notion that long duration of diabetes constitutes an independent risk factor for ED. Studies from Ethiopia [21] and Benin [33] have demonstrated that a diabetes duration exceeding 10 years is significantly associated with ED. Furthermore, a duration of diabetes > 5 years was identified as a risk factor for ED in a Sri Lanka study [31] and ≥ 49 months in a Chinese study [42]. In the present study, uncontrolled DM (Hb A1c ≥ 7%) was not significantly associated with ED. However, other studies have demonstrated that Hb A1c level of ≥ 7% is a risk factor for ED, as observed in Benin [33], Ethiopia [38] and India [43]. The lack of a significant association in the present study could be due to differences in the assessment tools used for glycaemic targets and the cutoff values used in each study.

In the present study, 44.9% of patients were found to have ASCVD, with CAS being the most common, followed by LLA, CHD and ischaemic stroke. Our previous study showed that the prevalence of CHD and LLA was 27.3% and 23%, respectively [6]. However, a study from Gabon reported that 63.4% of patients had macrovascular complications [25]. Studies conducted in Morocco (24%) [27] and Sri Lanka (9.2%) [31] found lower rates of ASCVD. This variability in prevalence can be attributed to differences in glycaemic control across studies, as well as variations in study settings (diabetology—endocrinology, cardiology, internal medicine, urology or general medicine departments), and the criteria used to diagnose these complications.

The present study demonstrated that ASCVD and CAS were significantly associated with ED, as evidenced by bivariate and multivariate analyses. The severity of ED was found to be significantly higher in these patients. This association has been supported in several other studies. In a study by Milama et al. in Gabon, the presence of macrovascular complications, LLA and CHD increased the risk of ED by 5.23, 7.32 and 3.69 times, respectively [25]. In the cohort of Banks et al. in Australia, men with severe ED had a significantly higher risk of CHD, heart failure, peripheral arterial disease, other cardiovascular diseases and all‐cause mortality [44]. Similarly, the ADVANCE study established a correlation between ED and an increased risk of cardiovascular incidents, CHD and cerebrovascular disease [45]. In a study conducted in India by Dsouza and Rahman, the presence of ED was identified as a predictive factor for CHD in patients with type 2 DM [46].

### 4.1 Strengths and Limitations of the Study

To our knowledge, the present study was one of the first conducted specifically on this topic in our country. This approach helped us consolidate the databases in our country and improve the care of our patients. It also encourages the initiation of national‐scale studies with large sample sizes.

However, the present study had limitations. The cross‐sectional study design only indicates a temporal association between dependent and independent variables. Certain important information was not included in all medical records, such as depression, testosterone, plasma vitamin D and therapeutic aspects of ED. These variables are known to be independent risk factors associated with ED. Therefore, the generalisability of the study findings is limited.

## 5 Conclusion

The prevalence of ED was found to be high and associated with ASCVD. Consequently, screening for ED should be strengthened in elderly individuals with other cardiovascular risk factors, those with type 2 and long duration of DM. The presence of ED suggests a need for investigation into the usual ASCVD, and vice versa. The primary preventive measure appears to be the management of other cardiovascular risk factors, which aims to reduce the incidence of ASCVD and ED. Additionally, a study validating the translation of the IIEF‐5 questionnaire into Malagasy is warranted.

## Author Contributions

**Sitraka Angelo Raharinavalona:** conceptualization, investigation, methodology, validation, software, data curation, formal analysis, project administration, resources, visualization, writing – review and editing, funding acquisition, writing – original draft. **Tafitarilova Dorland Ranjandriarison:** investigation, software, validation, visualization. **Thierry Razanamparany:** investigation, validation, formal analysis, visualization. **Romuald Randriamahavonjy:** supervision, writing – review and editing, project administration, visualization. **Andrianirina Dave Patrick Rakotomalala:** project administration, supervision, writing – review and editing, visualization.

## Conflicts of Interest

The authors declare no conflicts of interest.

## Data Availability

The data sets used and/or analysed during the current study are available from the corresponding author on reasonable request.
